# How well do malaria tests correlate with disease severity? Comparison of parasite density in children with mild and severe malaria

**DOI:** 10.5281/zenodo.10887755

**Published:** 2014-06-26

**Authors:** Sarah N. Kituyi, Nancy Nyakoe, Joseph N. Ngeranwa, Steven Runo, John N. Waitumbi

**Affiliations:** 1 Biochemistry and Biotechnology Department, Kenyatta University, Nairobi, Kenya; 2 Walter Reed Project, Kenya Medical Research Institute, Kisumu, Kenya.

## Abstract

**Background:**

Accurate diagnosis of malaria is key to proper management and control and an ideal diagnostic parameter that correlates to disease outcome is required. The former would be helpful in correctly identifying patients that need hospitalisation versus those that can be managed at home. This study determined how well the density estimates by microscopy, qPCR and *Pf*HRP-2 correlate to malaria severity.

**Materials and Methods:**

Patients aged ≤ 5 yrs with severe (n = 60, Hb ≤ 6 g/dl) and mild (n = 60, Hb > 6 g/dl) malaria were enrolled to take part in a case control study at Kisumu District Hospital, Western Kenya. Parasite load was determined by microscopy, qPCR targeting the *18s* rRNA gene and *Pf*HRP-2 antigen ELISA.

**Results:**

The median parasite load and the 25^th^ and the 75^th^ percentile by microscopy in children with severe malaria (SM) was 49,958 parasites/μl (12,013-128,695) compared to 24,233 (6,122-103,886) in the group with mild malaria (MM), P = 0.10. By qPCR, the translated median parasite density was 31,550 parasites/μl (4,106-196,640) in the SM group compared to 24,365 parasites/μl (5,512-93,401) in the MM group (P = 0.73). According to *Pf*HRP-2, the translated median parasite load in children with SM was 628,775 parasites/μl (332,222-1.165x106) compared to 150,453 (94,292-399,100) in children with MM (P < 0.0001).

**Conclusions:**

Unlike microscopy and qPCR, the parasite load detected by *Pf*HRP-2 correlates with disease severity. Because of its unique attributes, *Pf*HRP-2 is able to account for trophozoites and schizonts that are sequestered away from peripheral circulation. Because it persists in circulation, it also serves as an indicator of the magnitude of current and recent infections.

## 1 Introduction

Malaria is a serious global health problem, and ranks the third most common disease after HIV/AIDS and tuberculosis in Africa [1,2]. Infection is caused by five species of *Plasmodium* [3-5], which are transmitted through the bite of an infected female *Anopheles* mosquito [6,7]. The outcome of an infection depends on both host and parasite factors. Factors determining parasite prevalence and densities are, among others: the infecting species (for instance, *P. falciparum* infections are more severe [8] than *P. ovale*, *P*. *malariae* and *P. vivax*)*,* parasite virulence, multiplication rates and drug resistance profiles [9]. The host factors include general immune status [3], specific immunity to malaria, presence or absence of protective genetic traits (such as sickle cell anaemia, glucose-6-phosphate dehydrogenase (G6PD) deficiency and thalassemia) [10], age and pregnancy [8]. Degrees of anaemia and parasite density are the common indicators of malaria severity. Of these two, parasite density is the most useful because it is specific for malaria, but unfortunately, when evaluated by microscopy, it does not correlate with disease severity [11].

The testing of newly developed antimalarial drugs and vaccines requires a diagnostic method that is sensitive, specific and can quantify the parasite level [12,13]. In addition, a malaria diagnostic parameter that correlates with disease severity would add a third dimension to diagnosis, namely outcome prediction. This would also assist in triaging patients with severe malaria for admission and those with a predicted better outcome could be managed as outpatients. Prediction of the severity of the disease requires a diagnostic method that can indicate the total parasite load irrespective of their development stage and location [14]. Since parasitaemia estimated by microscopy fails to account for the more pathogenic sequestered parasite population [15], this study evaluated the suitability of alternative assays, including ELISA of *P. falciparum* histidine-rich protein-2 (*Pf*HRP-2), and quantitative polymerase chain reaction (qPCR) for the prediction of severe malaria.

## 2 Materials and Methods

### 2.1 Study design

This study was performed on samples collected in a hospital based case control study in children presenting at different times for admission at the Kisumu District Hospital in Western Kenya, a malaria holoendemic region. Sample preparation and subsequent analysis by microscopy, *Pf*HRP-2 ELISA and qPCR were performed at the Walter Reed Project/Kenya Medical Research Institute (WRP/KEMRI) laboratory in Kisumu, Kenya.

### 2.2 Study population

Children (age ≤ 5 years, n = 120) were enrolled at the Kisumu District Hospital paediatric ward. They were grouped into those with severe malaria (SM; n = 60) and matched by age and gender with others having mild malaria (MM; n = 60).

### 2.3 Inclusion and exclusion criteria

Participation of subjects in the group with severe malaria was guided by World Health Organization (WHO) specifications of severe malaria [16], such as the presence of asexual *P. falciparum* parasitaemia confirmed by a positive blood smear and anaemia (haemoglobin < 6 g/dl). For the group with mild malaria, inclusion in the study was based on the microscopic confirmation of a positive smear, haemoglobin levels > 6 g/dl and the absence of any other indicators of severe malaria.

Subjects were excluded from participation if there was evidence of concomitant infections (i.e., pneumonia, meningitis, sepsis, etc.), evidence of immune-compromised status (e.g., thrush and tuberculosis), and history of blood transfusion within the preceding six months. Children were also excluded if no consent from the parent was obtained and if they had a history of malignancy (e.g. Burkitt’s lymphoma), evidence of severe calorie imbalance (wasting < 70% of 50th percentile weight/height ratio for age) and evidence of protein deficiency (oedema, hair discoloration and abdominal distention).

### 2.4 Ethical consideration

This study was part of a large project conducted at the Walter Reed Project Kisumu. Ethical and scientific approval for the use of these samples was obtained from the Ethical Review Committee of the Kenya Medical Re-search Institute, Nairobi, Kenya (KEMRI SSC #879), and the Walter Reed Army Research Institute of Human Use Research Committee, Silver Spring, MD, USA (WRAIR #1145). Informed consent from the parents or guardians of the children was also obtained before enrolment of the children in the study. Patient data obtained in this study were handled confidentially and laboratory coding was used in the identification of the subjects.

### 2.5 Laboratory procedures

For the reported assays, sterile technique was used to collect 1 ml of whole blood with EDTA as anticoagulant. After thorough mixing, about 200 μl was used for counting total red blood cells with a Coulter counter, 200 μl for nucleic acid preparation, 500 μl for *Pf*HRP-2 and about 20 μl for microscopy blood smears. Whole blood was stored at -70°C until required. Before use, samples were thawed at room temperature and kept on ice during the entire as-say.

### 2.6 Microscopy

For Giemsa stained thick and thin smears, whole blood (6 μl) was used to make thick smears of about 10 mm diameter. A small drop of blood (2 μl) was used to make the thin smear and observed under 200× magnification high power fields for the presence of malaria parasites, the infecting species and the parasite numbers. For quantification of the parasite density in the thick smear, malaria parasites were counted against 200 white blood cells (WBCs). Using absolute quantification methods [17], the parasites counted against 200 WBCs were expressed as a fraction of 8000 WBCs to obtain the number of malaria parasites in 1 μl of blood. The parasite density in the thin film was determined by counting the number of infected red blood cells (RBCs) seen against 2000 RBCs.

### 2.7 Antigen detection by ELISA

Enzyme-linked immunoassays (ELISAs) were done using a commercial *Pf*HRP-2 ELISA kit (Malaria Ag CELISA; Cellabs Pty. Ltd., Brookvale, Australia). Each patient’s whole blood sample was tested using two monoclonal anti-bodies targeting different epitopes in a reaction setup in pre-coated anti-*P. falciparum* IgM capture antibody plates supplied alongside the kit as previously described [11]. The amount of bound antigen was read by spectrophotometry in the range of 450-650 nm using the SoftMax Pro 4.8 microplate software and reader (Molecular Devices, Sunnyvale, CA, USA). A standard curve was generated using a recombinant *Pf*HRP-2 antigen of known concentration of infected RBCs and used to determine the unknown concentration of each sample by finding the opposite concentration to the absorbance. The concentration range of the standard curve was 0.061-1.378.

### 2.8 Total Nucleic Acid Extraction

Total nucleic acids (RNA and DNA) were isolated from 200 μl of whole blood using a QIAmp MinElute Virus Spin blood kit (QIAGEN Inc., CA, USA) according to the manufacturer’s guidelines. The eluted total nucleic acids were stored in aliquots of 50 μl at -20°C until use.

### 2.9 PCR amplification of total nucleic acids

Amplification of the total nucleic acids was done by real time-quantitative PCR (RT-qPCR) using primers and probes specific for the *Plasmodium* 18s rRNA gene. The reactions were performed in a volume of 25 μl. Amplification and amplicon detection were done in an ABI Prism 7300 (Applied Biosystems) programmed to perform the following reaction cycles: an initial 50°C step for 30 min for reverse transcription, denaturing for 10 min at 95°C, 45 cycles at 95°C for 15 s, followed by annealing at 60°C for 1 min. Results were displayed in the form of amplification plots with Ct values, which were converted into raw para-site counts by extrapolation from a standard curve of known number of malaria infected RBCs.

### 2.10 Data analysis

The data generated were entered into Excel spreadsheets and analysed using Graph Pad Prism 5. The median parasitaemia levels and the median red blood cell count figure were compared between groups using a Mann-Whitney U test. The relationship between the parasite load by the *Pf*HRP-2 and qPCR assays to the gold standard was established by Pearson’s correlation analysis. All values were considered significant at P < 0.05.

**Figure 1.**
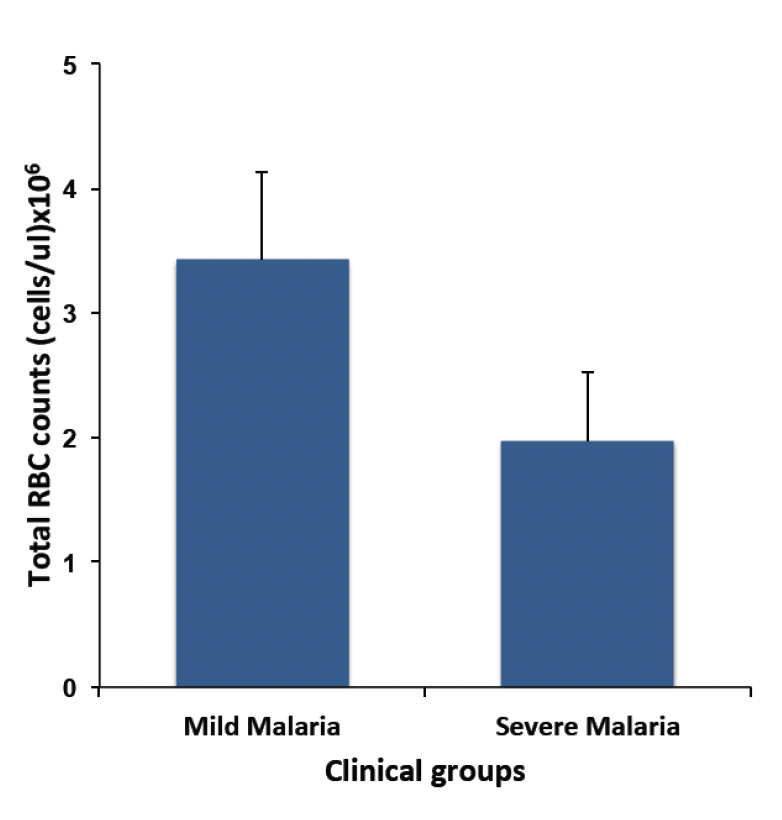
Comparison of the median total red blood cell counts between groups. Children with *falciparum* malaria (n = 56) were grouped into mild (n = 28) and severe malaria (n = 28). Comparison of the median between groups was done using the Mann-Whitney U test. Data presented as median (bars). Difference be-tween both groups: P<0.001.

## 3 Results

### 3.1 Parasite density by diagnostic techniques

The data were analysed by pairing each severe malaria case (n = 28) to the age- and gender-matched mild malaria control group (n = 28), which prompted the exclusion of samples without exact matches. The median and the 25th and 75th percentiles of parasite load in each clinical group (n = 28) by the three diagnostic methods were analysed by Mann-Whitney U test. As shown in Table 1, the median parasite density and the 25th and the 75th percentile by microscopy in the SM cases was 49,958 parasites/μl (12,013-128,695) compared with 24,233 parasites/μl (6,122-103,886) in the MM controls. These densities were not statistically different (P = 0.103). The parasite biomass by *Pf*HRP-2 assay depicted higher median parasite numbers in both groups: 628,775 parasites/μl (332,222-1.165x106) for the SM group and 150,453 parasites/μl (94,292-399,100) for the MM group (P < 0.0001). The median parasite density and the 25th and the 75th percentile by qPCR for SM cases was 31,550 parasites/μl (4,106-196,640) compared with 24,365 parasites/μl (5,512-93,401) for MM controls (P = 0.7336).

**Table 1.** Malaria parasite density between clinical groups by microscopy, *Pf*HRP-2, *P*LDH and qPCR. Data are presented as median (25^th^ and 75^th^ percentile). Children with *falciparum* malaria (n = 56) were grouped into mild (n = 28) and severe malaria (n = 28). Comparison of the median values was determined using the Mann-Whitney U test.

Diagnostic method	Mild malaria (n = 28) Median (25^th^-75^th^)	Severe malaria (n = 28) Median (25^th^-75^th^)	P value
Microscopy	24,233	49,958	0.103
	(6,122-103,886)	(12,013-128,695)	
*Pf*HRP2	150,453	628,775	<0.0001
	(94,292-399,100)	(332,222-1.165x10^6^)	
qPCR	24,365	31,550	0.7336
	(5,512 – 93,401)	(3,743 – 203,969)	

### 3.2 Correlation of the parasite load by *Pf*HRP-2 and qPCR with parasite density by microscopy

Because microscopy is the gold standard for the diagnosis of malaria, the correlation between parasite density by microscopy and the parasite load by *Pf*HRP-2 and qPCR was measured by Pearson’s correlation analysis. This analysis illustrated a positive correlation between the parasite loads measured by *Pf*HRP-2 (r = 0.585, P = 0.001**)** and qPCR (r = 0.527, P = 0.012) assays to the parasite density depicted by microscopy in the MM group, which was statistically significant (Table 2). However, in the SM group, the correlation between parasite density by the HRP-2 assay and parasite density by microscopy was the poorest and not statistically significant (r = 0.336, P = 0.080). The qPCR assay depicted the best positive correlation to para-site density by microscopy in the SM group (r = 0.780, P < 0.0001).

**Table 2.** Association of the parasite load by diagnostic techniques and parasite density by microscopy. The association between the parasite loads by the other diagnostic methods and the parasite load depicted by microscopy was measured by Pearson’s correlation analysis. Data (n = 28) in each group presented using Pearson’s r-values and P values at α = 0.05.

Parameter	Parasite density by microscopy			
	Mild malaria (A)		Severe malaria (B)	
	r	P	r	P
*P*LDH	0.527	0.004	0.558	0.002
*Pf*HRP-2	0.585	0.001	0.336	0.080
qPCR	0.527	0.012	0.780 <	0.0001

## 4 Discussion

In this study, parasite densities by three methods of diagnosing malaria (microscopy, *Pf*HRP-2 and qPCR) were evaluated as to how well they correlated to malaria severity. When the parasite loads measured by microscopy, anti-gen detection and qPCR were compared between the two clinical groups, the density by each method was higher in SM cases compared with controls. However, a comparison of the median parasite densities indicated significant differences only when the analysis was done by the *Pf*HRP-2 assay (P < 0.0001). The *Pf*HRP-2 assay had the highest median parasite densities (150,453 parasites/μl in the MM group and 628,775 parasites/μl in the SM group). The superiority of the *Pf*HRP-2 assay over the other assays is attributed to the production of large numbers of *Pf*HRP-2 copies into circulation by parasites in peripheral circulation and by those sequestered in organs away from circulation. Unlike the other assays, the parasite load by the *Pf*HRP-2 assay is a reflection of the total parasite biomass that may have accumulated from both past and current infections. Previous studies have also indicated that the *Pf*HRP-2 assay is superior for the diagnosis of *P. falciparum* malaria [18]. This might explain the high median parasite density observed in the *Pf*HRP2 assay in this study because *P. falciparum* is the most prevalent malaria para-site in the region [19].

Because microscopy is considered the gold standard for malaria diagnosis, the correlation of parasite density by microscopy to the parasite density by the *Pf*HRP-2 and qPCR was determined by Pearson’s correlation analysis. The data showed a positive correlation between the para-site loads measured by all methods to the parasite density depicted by microscopy in the MM group (*Pf*HRP-2: r = 0.585, P = 0.001; qPCR: r = 0.527, P = 0.012). However, in the SM group, the correlation of the parasite density by the *Pf*HRP-2 assay was the poorest (r = 0.336 *P* = 0.08). This is an aspect that has been demonstrated in previous studies that also identified a very poor association between the two [11,18]. The lack of correlation can be attributed to many factors, such as the persistence of the *Pf*HRP-2 antigen in circulation, and the production of very high quantities of this protein [11]. The latter makes it difficult to relate the numbers of the parasites seen in peripheral circulation by microscopy to the numerous antigens detected in a *Pf*HRP-2 assay. Unlike microscopy, the parasite density by the *Pf*HRP-2 assay in the SM group reflects an accumulation from past and current infections emerging from the parasites in circulation and those sequestered away from circulation. The qPCR assay depicted the best positive correlation to the parasite density by microscopy in the SM group (r = 0.780, P < 0.0001). Previous studies had assumed that the high sensitivity of PCR in malaria diagnosis was due to remnant DNA from phagocytised parasites and low levels of peripheral parasitaemia [20], but the correlation found in this study would dispute these findings.

The development of SMA in young children is an important clinical outcome of an infection with malaria para-sites. Therefore, comparison of the ability of different diagnostic methods to predict the development of severe malaria anaemia in children is vital for triaging patients for the right case management and treatment. This study was not designed to evaluate this association because SMA was used as the basis of inclusion of subjects in the group with severe malaria, but comparison of the total RBC counts between groups did confirm that children with SM had low RBC counts.

Accurate diagnosis of malaria is key to effective treatment and control, and delayed diagnosis increases morbidity and mortality [3]. Ideally, the best diagnostic techniques should accurately indicate the presence or absence of malaria parasites, identify the infecting species, quantify the parasite count and measure the efficacy of antimalarial therapy [13]. Because numerous studies have questioned the ability of microscopy (the current gold standard) to correctly perform these functions, the evaluation of the ability of alternative methods for the same purpose as depicted in this study is considered important.

## 5 Conclusions

This study compared the ability of malaria diagnostic methods to quantify the parasite load and to correctly distinguish cases of severe and mild malaria. In addition, the study describes how the parasite load by each diagnostic method correlates to the parasite density as measured by the gold standard. We showed that, unlike microscopy and qPCR, the parasite load detected by *Pf*HRP-2 correlates well with disease severity and can be used to distinguish severe from mild malaria.
